# Lethal Thermal Impact at Periphery of Pyroclastic Surges: Evidences at Pompeii

**DOI:** 10.1371/journal.pone.0011127

**Published:** 2010-06-15

**Authors:** Giuseppe Mastrolorenzo, Pierpaolo Petrone, Lucia Pappalardo, Fabio M. Guarino

**Affiliations:** 1 Istituto Nazionale di Geofisica e Vulcanologia, sezione di Napoli, Osservatorio Vesuviano, Napoli, Italy; 2 Museo di Antropologia, Centro Musei delle Scienze Naturali, Università degli Studi di Napoli Federico II, Naples, Italy; 3 Dipartimento di Biologia Strutturale e Funzionale, Università degli Studi di Napoli Federico II, Naples, Italy; German Cancer Research Center, Germany

## Abstract

**Background:**

The evaluation of mortality of pyroclastic surges and flows (PDCs) produced by explosive eruptions is a major goal in risk assessment and mitigation, particularly in distal reaches of flows that are often heavily urbanized. Pompeii and the nearby archaeological sites preserve the most complete set of evidence of the 79 AD catastrophic eruption recording its effects on structures and people.

**Methodology/Principal Findings:**

Here we investigate the causes of mortality in PDCs at Pompeii and surroundings on the bases of a multidisciplinary volcanological and bio-anthropological study. Field and laboratory study of the eruption products and victims merged with numerical simulations and experiments indicate that heat was the main cause of death of people, heretofore supposed to have died by ash suffocation. Our results show that exposure to at least 250°C hot surges at a distance of 10 kilometres from the vent was sufficient to cause instant death, even if people were sheltered within buildings. Despite the fact that impact force and exposure time to dusty gas declined toward PDCs periphery up to the survival conditions, lethal temperatures were maintained up to the PDCs extreme depositional limits.

**Conclusions/Significance:**

This evidence indicates that the risk in flow marginal zones could be underestimated by simply assuming that very thin distal deposits, resulting from PDCs with poor total particle load, correspond to negligible effects. Therefore our findings are essential for hazard plans development and for actions aimed to risk mitigation at Vesuvius and other explosive volcanoes.

## Introduction

Pyroclastic density currents (PDCs), turbulent hot mixtures of fine ash and gas flowing down volcano slopes at high speeds, are common in volcanic explosive eruptions. They can devastate large areas and cause numerous fatalities by exposure to mechanical impact, extreme heat and dusty gas [1].

In proximal and intermediate PDCs emplacement zone, all these factors commonly exceed the critical threshold for human survival, thus indicating that none mitigation strategy is suitable except for the timely evacuation [2]. However, since the lethality of PDCs may decline up to human survival conditions with increasing distance, the evaluation of PDCs effects near the flow termination is widely debated being crucial for risk mitigation management, particularly for distal areas often densely inhabited. Nevertheless, these aspects are documented only in a few modern eruptions and difficult to retrieve from historical record, due to scarce availability of data on thin distal layers.

A unique, although poorly-investigated, record of the far-reaching lethal effects of PDCs on people and the environment is well preserved at the distal archaeological site of Pompeii, ca. 10 kilometres southeast of Vesuvius. Here, the remains of hundreds human victims were discovered within the pyroclastic surges of the 79 AD eruption [3], [4]. In order to evaluate the potential effects of a future Plinian eruption at Vesuvius we have assessed the PDCs overall impact by combining volcanological and bio-anthropological studies with experiments and numerical modeling.

## Materials and Methods

No ethics approval was required to the Ethics Committee (Comitato Etico per le Attività Biomediche dell'Università degli Studi Federico II di Napoli”, Azienda Ospedaliera Universitaria della Seconda Università degli Studi di Napoli, Naples, Italy) because human and animal (Equus caballus) bone samples used in this work are Institute certified specimens (sample inventory N. 14083/880/71 of the Department of Structural and Functional Biology and sample inventory N. 2408/Z653/50 of the Museum of Zoology) at the University of Naples “Federico II”. Samples were officially provided for our study by informed written consent from the University of Naples “Federico II”.

### PDCs physical model

The physical model adopted in our numerical simulations of PDCs is described by the equations of uniform flow for a Bingham or Newtonian fluid [5], [6].

The steady, uniform velocity profile for a Bingham flow in infinitely wide channels is given by:
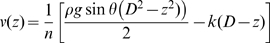
where z≥Dc is the height (measured from the bottom of the channel), k is yield strength, ρ is the density of flow material, g is acceleration due to of gravity, θ is the slope of ground, η is the viscosity, D the total flow depth, and Dc is the plug thickness,
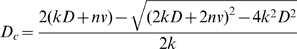
The acceleration of the plug in a wide channel is [5]:
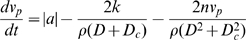
where vp is plug velocity and |a| is the modulus of the gravity contribution to the flow motion.

Resistance terms in the equation describing a Bingham flow unit depends on several factors. For a Bingham flow, the transition from laminar to turbulent flow depends upon two dimensionless numbers, the Reynolds number Re = ρv D/η, and the Bingham number Bi = k D/η v. From empirical relations, Middleton and Southard [7] observed that, when Bi exceeds about 1.0 the onset of turbulence occurs for Re/Bi≥1000. According to McEwen and Malin [5], we the frontal air drag is assumed to be negligible, so that the deceleration due to air drag on the upper surface [8] of the flow is:
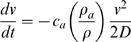
with ca ranging between 0.1 and 1.

### Light and Scanning electron microscope

We examined undecalcified and unstained ground sections, and decalcified and stained cryostat sections of bone samples. Ground sections (80–100 µm thick) were prepared after embedding in LY-554 araldite resin (Vantico) and observed under light microscopy, in ordinary and polarized light. Bone cryostat sections (7 µm thick) were obtained after decalcification in 3% nitric acid from 2 to 4 h, depending on the size of bone samples and their brittleness. These sections were stained by thionin (Sigma) and 4′,6′-diamidino-2-phenylindole (DAPI, Sigma) to investigate the persistence of DNA within osteocyte lacunae [9]. DAPI-stained sections were observed under fluorescence light microscopy. Concerning SEM study a bone slice, adjacent to that used for light microscopy, was examined using Cambridge 250 Mark 3 scanning electron microscope, working at 20 kV beam voltage.

### Laboratory heating experiments

Recent human bone samples were exposed at temperatures progressively ranging from 100°C to 800°C, using a Linkam TS 1500 system, at a constant rate of 100°C/min. For each test, the specimen was maintained at a first rate temperature of 60″ and the final one of 30″. As further control, fresh femur bone samples of recent horse (*Equus caballus*) were also tested, with rates temperature of 60″ to 180″. Bone colours of the experimentally heated specimens were assessed by comparison to Munsell [10] soil color chart.

## Results

### Pyroclastic deposit features and numerical modeling

The 79 AD Vesuvius eruption generated a sequence of six distinctive pyroclastic surges (S1 to S6) and flows with increasing power, which caused landscape modification as well widespread building collapse and fatalities [3]. The resulting ash deposits have thicknesses ranging from tens of metres near the vent to few millimetres at the flow periphery. In particular, the early three surges (S1 to S3) stopped ahead the northwestern walls of Pompeii, while the later ones (S4 to S6) over passed the town. The last two surges traveled up to a distance even exceeding 15 kilometres from the vent, whereas the S4 surge deposit least traces are confined within a few hundred of metres next to the south and southeastern walls.

The S4 pyroclastic surge is definitely relevant, since caused most of the fatalities in Pompeii in spite of the only 3 centimetres thick resulting deposit, a covered area of ca. 80 km^2^ and a modest total mass that we estimate in the order of 2×10^9^ kg, at least one order of magnitude less than the overlying S5 and S6 surges. Textural and granulometric analyses of S4 deposit reveal absence of vertical grain-size grading with a graphic mean of 175 microns (Mdφ = 2.5) and graphical standard deviation of 300 microns (σφ = 1.9). This indicates that ash emplaced suddenly in a single depositional event resulting from dusty gas mixture deflation in response to horizontal velocity and turbulence dumping at the flow termination.

**Figure 1:**
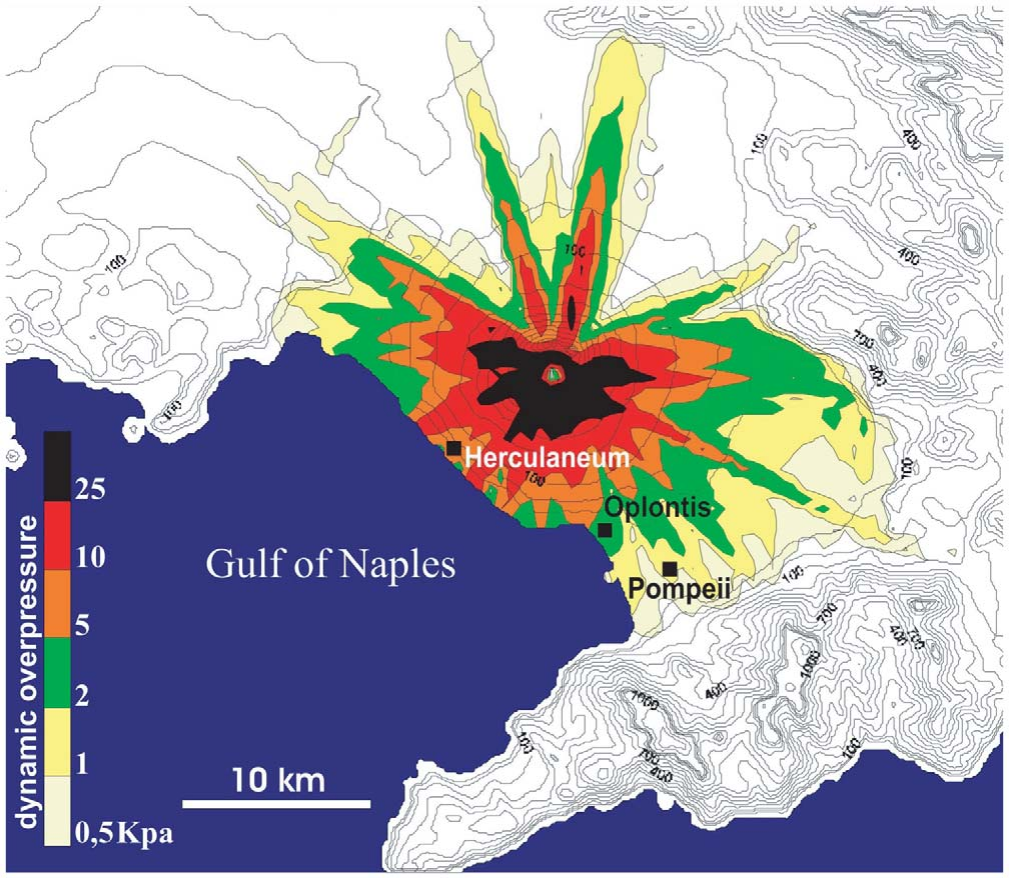
Numerical simulation of S4 PDC. Areal distribution and dynamic overpressure at Vesuvius of a pyroclastic surge cloud that is an analogue to the 79 AD upper main surges. Initial velocity = 50÷100 m/s, thickness = 30÷130 m, density = 2÷50 kg/m^3^, viscosity = 2×10 −5 Pa/s, yield strength = 0 Pa (scale bar values in KPa). The numerical simulation is based on a simple model of a gravity-driven pyroclastic current that stops by en masse freezing.

In order to investigate dynamics and emplacement mechanisms of S4 PDC at Pompeii, we have developed a numerical model (Figure 1). It has been retrieved by a Bayesian data inversion [11] of a set of 300 numerical simulations on a Somma-Vesuvius and surrounding plains Digital Elevation Model, produced by sampling a wide matrix of input parameters typical of Vesuvius PDCs [12]. Field data such as flow depositional limits as well as the computed PDC velocity, density and thickness relative to the outcrops of Osservatorio Vesuviano (northern *Herculaneum* district), *Oplontis* and Pompeii, at ca. 3, 7 and 10 kilometres from the vent respectively, have been used to constrain the inversion. These parameters have been computed from the measured deposits thickness, grain size distributions and components density by developing an original code using Matlab environment for sedimentation mechanisms of stratified pyroclastic flows [13].

We calculated that in *Herculaneum*, *Oplontis* and Pompeii the PDC advanced with average density of ca. 10, 5 e 1.5 kg/m^3^ and as thick as ca. 60, 25, 18 metres, with velocity of ca. 45, 28, 29 m/s, respectively. The best PDC model obtained with the above constrains is a flow with Newtonian rheology, with a velocity at the vent of 50÷100 m/s, front thickness of 30÷130 metres, density of 1.5÷50 kg/m^3^ and average run-out within 10 kilometres. The inferred PDC mass of ca. 7.5×10^7^ kg which emplaced behind Pompeii, divided by the total mass flow rate through the town ranging from 1.5×10^5^ to 7.4×10^6^ kg/s (equivalent to 26÷240 kg/s/m^2^, of ca. 110 kg/s/m^2^ in average) computed from the above results, provides the passage time of PDC cloud between ca. 30 and ca. 1.5×10^2^ seconds.

These results indicate that the pyroclastic surge advanced as a dilute turbulent poorly-energetic deflating cloud and emplaced suddenly, also lead by town buildings and walls and trench barrier effect. Such behavior accounts for the lack of evidence of mechanical impact on structures and of engulfment and transport in the S4 deposit of stuff such as tiles and bricks.

### Site and laboratory bioanthropological evidence

In order to recognize the effects on people of the S4 surge, we analyzed the human victims remains from the Pompeii archeological site. Here, within the lapilli bed were found 394 skeletons of victims of the early fallout eruptive phase, 90% of whom died within buildings probably due to roof and floor collapse. Deposits of the later S4 surge preserved the remains of 650 victims heretofore supposed to have died by ash suffocation [4], [14], [15].

We studied the body postures of 93 well-preserved plaster casts of the surge victims at Pompeii (Table 1). For comparison we also examined 37 additional corpses of surge fatalities at *Oplontis* (Table 2), a Roman seaside suburban site located ca. 2 kilometres west-northwest of Pompeii, and 78 skeletons of surge victims unearthed at *Herculaneum* (Table 3) [16]. The body postures assumed at the time of PDC emplacement were assessed by both direct analysis of casts and skeletons as well as by archive photographs, to search for evidence of PDCs lethal effects caused by mechanical impact, heat exposure and dusty gas inhalation in victims found inside as well outside buildings.

**Table 1:** Types and occurrence of postures detected on 93 human victims from Pompeii.

N°	Specimen	Sex	Age class	type a	type b	type c	type d	type e	N°	Specimen	Sex	Age class	type a	type b	type c	type d	type e
1	PO-CCR1	F	AD	X	0	0	X	X	48	PO-SPN6	F?	AD	0	X	0	0?	X
2	PO-CCR2	F?	AD	X	0	0	X	X	49	PO-SPN7	M	AD	X	0	X?	X	X
3	PO-CCR3	//	Sad	0	X	0	0?	X	50	PO-SPN8	//	Sad	X	0	0	X	X
4	PO-CCR4	//	AD	X	0	0	0?	X	51	PO-SPN9	//	Sad	0	X	0	0?	X
5	PO-CCR5	//	AD	0	X	0	0?	X	52	PO-SPN10	//	Sad	X	0	0	X	X
6	PO-CCR6	M	AD	X	0	X?	X	X	53	PO-SPN11	//	AD	X	0	0	X	0?
7	PO-CCR7	//	AD	0	X	0	X	X	54	PO-SPN12	M	AD	X	0	0	X	X
8	PO-CCR8	//	AD	X	0	0	X	//	55	PO-SPN13	F?	AD	X	0	0	0?	X
9	PO-CCR9	M	Sad	0	X	0	X	0?	56	PO-SPN14	M?	AD	X	0	0	X	X
10	PO-FOR1	M	AD	X	0	0	X	X	57	PO-SPN15	//	Sad	X	0	0	X	X
11	PO-FOR2	M	AD	X	0	0	X	X	58	PO-ISW1	M?	AD	X	0	0	X	X
12	PO-CPR1	M	AD	0	X	0	X	0?	59	PO-ISW2	M?	AD	X	0	0	X	X
13	PO-CPR2	M	AD	0	X	0	0?	X	60	PO-ISW3	//	AD	X	0	0	0?	X
14	PO-CPR3	M	AD	X	0	0	X	X	61	PO-ISW4	//	AD	X	0	0	X	X
15	PO-CBO1	F	AD	X	0	0	X	X	62	PO-ISW5	//	AD	X	0	0	X	X
16	PO-CBO2	//	Sad	X	0	0	X	X	63	PO-ISW6	//	AD	X	0	0	0?	X
17	PO-CBO3	M	AD	X	0	0	X	X	64	PO-ODF1	F?	AD	0	X	0	0?	0?
18	PO-CBO4	M	Sad	X	0	0	X	X	65	PO-ODF2	//	Sad	0	X	0	X	0?
19	PO-CDL1	//	AD	0	X	0	X	0?	66	PO-ODF3	//	Sad	X	0	0	0?	X
20	PO-ANT1	M	AD	0	X	0	X	0?	67	PO-ODF4	M	AD	X	0	0	X	X
21	PO-ANT2	M	AD	X	0	0	X	X	68	PO-ODF5	M?	AD	X	0	0	X	X
22	PO-ANT3	//	AD	X	0	0	X	X	69	PO-ODF6	F	Sad	0	X	0	X	X
23	PO-ANT4	M	AD	X	0	0	X	X	70	PO-ODF7	F	AD	X	0	X?	X	X
24	PO-ANT5	M?	AD	X	0	0	X	X	71	PO-ODF8	//	AD	X	0	0	0?	X
25	PO-VDM1	M	AD	0	X	0	X	X	72	PO-ODF9	F?	AD	X	0	0	X	X
26	PO-VDM2	M	Sad	X	0	0	X	X	73	PO-ODF10	F?	AD	X	0	0	X	X
27	PO-DFR1	M	AD	X	0	0	X	X	74	PO-ODF11	F	AD	X	0	X?	X	X
28	PO-DFR2	F?	AD	X	0	0	X	X	75	PO-ODF12	//	Sad	0	X	0	X	X
29	PO-DFR3	M?	AD	X	0	0	X	0?	76	PO-ODF13	M	AD	X	0	0	X	X
30	PO-VDS1	M?	AD	X	0	0	X	X	77	PO-LGP1	//	AD	X	0	0	0?	X
31	PO-VDS2	//	AD	X	0	0	X	X	78	PO-CDM1	//	AD	X	0	0	0?	X
32	PO-PNC1	M	AD	X	0	0	X	X	79	PO-CDM2	//	AD	X	0	0	X	X
33	PO-PNL1	//	AD	0	X	0	X	X	80	PO-CDM3	//	AD	X	0	0	X	X
34	PO-PNL2	F	AD	0	X	0	0?	0?	81	PO-VCC1	//	AD	X	0	0	0?	X
35	PO-PNL3	M	AD	X	0	0	0?	X	82	PO-DEF1	//	AD	X	0	0	0?	X
36	PO-CDP1	M	AD	X	0	0	X	X	83	PO-GPA1	//	Sad	0	X	0	0?	0?
37	PO-CDP2	M	AD	X	0	0	X	X	84	PO-NMR1	//	Sad	X	0	0	0?	X
38	PO-CDP3	F	AD	0	X	0	X	X	85	PO-VDA1	//	AD	X	0	0	X	0?
39	PO-CDP4	M	AD	0	X	0	X	X	86	PO-CTV1	//	AD	X	0	0	0?	X
40	PO-CDP5	M	AD	X	0	0	X	X	87	PO-CTV2	//	AD	X	0	0	0?	X
41	PO-OCM1	M	AD	0	X	0	0?	0?	88	PO-CTV3	//	AD	X	0	0	0?	X
42	PO-OCM2	M	AD	X	0	0	X	X	89	PO-CTV4	//	AD	X	0	0	0?	X
43	PO-SPN1	F	AD	X	0	0	X	X	90	PO-CGP1	//	AD	0	X	0	0?	0?
44	PO-SPN2	//	AD	X	0	0	0?	X	91	PO-CGP2	//	AD	X	0	0	0?	0?
45	PO-SPN3	//	Sad	0	X	0	0?	X	92	PO-MAN1	//	Sad	X	0	0	X	X
46	PO-SPN4	M?	AD	X	0	0	X	X	93	PO-MAN2	M?	AD	X	0	0	X	X
47	PO-SPN5	//	Sad	0	X	0	X	X	**93**	**Pompeii**	**36/15**	**75/18**	**74%**	**26%**	**4%**	**69%**	**85%**

Type a = “*life-like*” stance; type b = “*sleep-like*” stance; type c = “*impact-like*” stance; type d = “*limb contraction*” stance; type e = “*pugilistic attitude*”; M = Male; F = Female; AD = Adult; Sad = Subadult.

**Table 2:** Types and occurrence of postures detected on 37 human victims from Oplontis.

N°	Specimen	Sex	Age class	type a	type b	type c	type d	type e	N°	Specimen	Sex	Age class	type a	type b	type c	type d	type e
94	OP-1-I15	M	AD	X	0	0	X	//	113	OP-20-I36	//	AD	X	0	0	0?	X
95	OP-2-I19	//	AD	X	0	0	0?	X	114	OP-21-I39	//	AD	X	0	0	0?	X
96	OP-3-I22	//	//	0	X	0	X	X	115	OP-22-I40	//	AD	X	0	0	X	X
97	OP-4-I29	//	//	0	X	0	//	//	116	OP-23-I41	//	Sad	X	0	0	X	X
98	OP-5-I31	M	AD	X	0	0	//	X	117	OP-24-I42	//	AD	X	0	0	X	X
99	OP-6-I32	//	AD	0	X	0	//	//	118	OP-25-I43	//	AD	X	0	0	0?	X
100	OP-7-I34	//	AD	X	0	0	X	X	119	OP-26-I44	//	AD	X	0	0	0?	X
101	OP-8-IE	//	AD	X	0	0	X	X	120	OP-27-I45	//	AD	X	0	0	X	X
102	OP-9-IF	M?	AD	X	0	0	X	X	121	OP-28-I46	//	Sad	0	X	0	0?	0?
103	OP-10-IG	//	Sad	X	0	0	X	X	122	OP-29-I47	//	Sad	0	X	0	0?	0?
104	OP-11-IH	//	AD	X	0	0	X	X	123	OP-30-I48	//	Sad	X	0	0	0?	0?
105	OP-12-II	//	AD	X	0	0	//	//	124	OP-31-I49	//	AD	X	0	0	X	X
106	OP-13-IL	//	AD	X	0	0	//	X	125	OP-32-I51	//	Sad	0	X	0	0?	0?
107	OP-14-IM	//	AD	X	0	0	X	X	126	OP-33-I52	//	AD	X	0	0	0?	X
108	OP-15-IN	//	AD	X	0	0	//	X	127	OP-34-IA	//	AD	X	0	0	//	X
109	OP-16-INB	//	AD	0	X	0	//	X	128	OP-35-IB	//	AD	X	0	0	//	X
110	OP-17-IO	//	AD	X	0	0	X	X	129	OP-36-IC	//	AD	X	0	0	//	//
111	OP-18-IP	F?	Sad	0	X	0	X	X	130	OP-37-ID	//	AD	0	X	0	X	//
112	OP-19-I35	//	AD	0	X	0	X	X	**37**	**Oplontis**	3/1	28/7	**73%**	**27%**	**0%**	**63%**	**87%**

Type a = “*life-like*” stance; type b = “*sleep-like*” stance; type c = “*impact-like*” stance; type d = “*limb contraction*” stance; type e = “*pugilistic attitude*”; M = Male; F = Female; AD = Adult; Sad = Subadult.

**Table 3:** Types and occurrence of postures detected on 78 human victims from Herculaneum, and total count for the Pompeii area.

N°	Specimen	Sex	Age class	type a	type b	type c	type d	type e	N°	Specimen	Sex	Age class	type a	type b	type c	type d	type e
131	ER-F5-1	M	Sad	X	0	0	X	0	171	ER-F10-36	M	Sad	X	0	//	X	0
132	ER-F5-2	F	AD	0	X	0	X	0	172	ER-F10-37	//	AD	X	0	//	0?	0
133	ER-F5-3	M	AD	X	0	0	X	0	173	ER-F10-38	//	Sad	X	0	//	//	0
134	ER-F10-1	M?	AD	X	0	0	//	0	174	ER-F10-39	M	Sad	0	X	//	X	0
135	ER-F10-2	M	Sad	0	X	0	X	0	175	ER-F10-40	?	Sad	0	X	//	//	0
136	ER-F10-3	M	AD	X	0	0	0?	0	176	ER-F10-41	F?	Sad	X	0	0	X	X
137	ER-F10-4	F	AD	X	0	0	0?	X	177	ER-F12-1	M?	Sad	X	0	0	0?	0
138	ER-F10-5	M	AD	X	0	0	//	0	178	ER-F12-2	F	AD	X	0	0	X	X
139	ER-F10-6	M	AD	X	0	0	X	0	179	ER-F12-3	F	AD	X	0	0	X	0
140	ER-F10-7	M	AD	0	X	0	X	0	180	ER-F12-4	M	AD	X	0	0	0?	0
141	ER-F10-8	M	Sad	0	X	0	X	0	181	ER-F12-5	M?	Sad	X	0	0	X	0
142	ER-F10-9	M	Sad	X	0	0	X	0	182	ER-F12-6	F?	Sad	0	X	0	X	0
143	ER-F10-10	M	AD	0	X	0	X	0	183	ER-F12-7	M?	Sad	0	X	0	X	0
144	ER-F10-11a	F	AD	X	0	0	X	0	184	ER-F12-8	M?	AD	X	0	0	//	0
145	ER-F10-11b	M	AD	0	X	//	//	0	185	ER-F12-9	F	AD	X	0	0	X	0
146	ER-F10-12	M	AD	X	0	0	X	0	186	ER-F12-10	M?	Sad	0	X	0	X	0
147	ER-F10-13	M	AD	X	0	0	X	0	187	ER-F12-11	M	AD	X	0	0	X	X
148	ER-F10-14	M	AD	0	X	0	0?	0	188	ER-F12-12	M?	Sad	X	0	0	//	0
149	ER-F10-15	F	AD	X	0	0	X	0	189	ER-F12-13	F?	AD	X	0	0	X	0
150	ER-F10-16	F	AD	X	0	0	X	X	190	ER-F12-14	M	Sad	X	0	0	X	0
151	ER-F10-17	M	AD	X	0	0	X	0	191	ER-F12-15	F	AD	X	0	0	X	X
152	ER-F10-18	F	AD	X	0	0	X	0	192	ER-F12-16	M	AD	X	0	0	X	0
153	ER-F10-19	M	AD	X	0	0	//	0	193	ER-F12-17	//	Sad	X	0	0	X	0
154	ER-F10-20	M	AD	X	0	0	0?	X	194	ER-F12-18	F?	Sad	X	0	0	X	0
155	ER-F10-21	M	AD	X	0	0	X	X	195	ER-F12-19	M	AD	X	0	0	X	0
156	ER-F10-22	M	AD	X	0	0	X	0	196	ER-F12-20	M	AD	0	X	0	X	0
157	ER-F10-23	M	AD	X	0	0	//	X	197	ER-F12-21	F	AD	X	0	//	//	0
158	ER-F10-24	F	AD	X	0	0	X	X	198	ER-F12-22	M	AD	X	0	0	X	0
159	ER-F10-25a	M?	AD	X	0	//	X	0	199	ER-F12-23	M	AD	X	0	0	X	0
160	ER-F10-25b	//	AD	0	X	//	X	0	200	ER-F12-24	M?	Sad	X	0	0	X	0
161	ER-F10-26	M?	Sad	0	X	0	//	0	201	ER-F12-25	M	Sad	X	0	0	X	0
162	ER-F10-27	F?	Sad	X	0	//	//	0	202	ER-F12-26	M	AD	X	0	0	X	0
163	ER-F10-28	F	AD	X	0	0	X	0	203	ER-F12-27	M	AD	X	0	0	X	0
164	ER-F10-29	F	AD	X	0	0	//	0	204	ER-F12-28	F	AD	X	0	//	X	0
165	ER-F10-30	M?	Sad	0	X	0	//	X	205	ER-F12-29	F?	Sad	X	0	0	X	0
166	ER-F10-31	//	AD	0	X	//	//	0	206	ER-F12-30	F	Sad	X	0	0	X	0
167	ER-F10-32	M?	Sad	0	X	0	X	0	207	ER-F12-31	F?	AD	X	0	//	//	0
168	ER-F10-33	F	Sad	0	X	//	//	0	208	ER-F12-32	//	Sad	0	X	0	X	0
169	ER-F10-34	F?	Sad	0	X	//	//	0	78	Herculaneum	46/24	49/29	72%	28%	0%	94%	20%
170	ER-F10-35	M	AD	0	X	//	//	0	208	Total sites	85/40	152/54	73%	27%	2.1%	76%	64%

Type a = “*life-like*” stance; type b = “*sleep-like*” stance; type c = “*impact-like*” stance; type d = “*limb contraction*” stance; type e = “*pugilistic attitude*”; M = Male; F = Female; AD = Adult; Sad = Subadult.

In Pompeii, most of the groups of victims display a variety of postures that can be arranged as 1) primary postures assumed at the time of death or 2) secondary, post-mortem postures.

**Figure 2:**
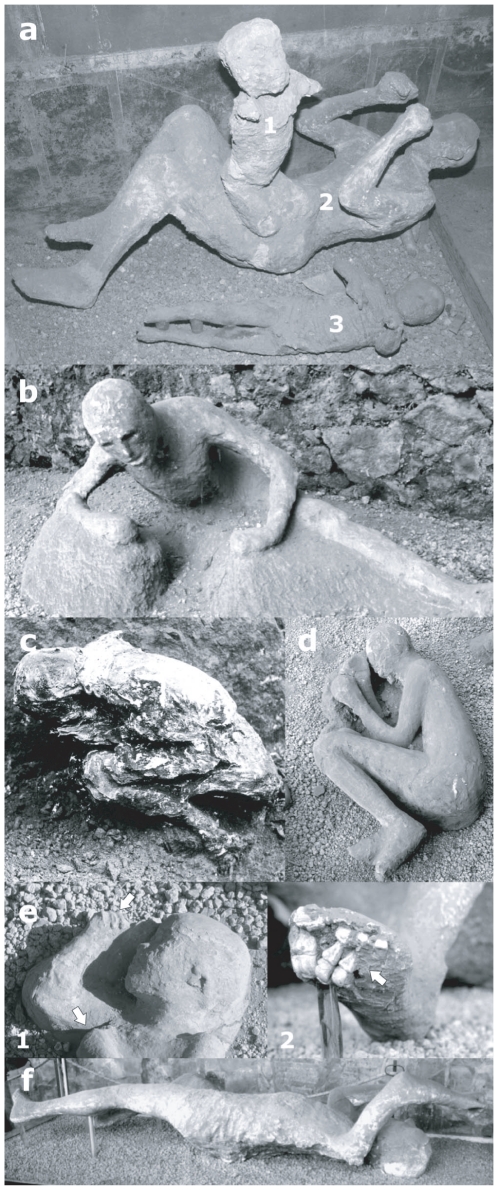
Typical body postures assumed by human victims in PDCs at Pompeii. “*Life-like*” stance: a^1^. infant and a^2^. adult female (House of the gold bracelet, Regio VI, Insula 17 [Insula Occidentalis], 42), b. adult male (The garden of the fugitives, Regio I, Insula 21), c. adult male (The Great Palaestra, Regio II, Insula 7); “*sleep-like*” stance: d. adult male (The garden of the fugitives, Regio I, Insula 21); “*limb contraction*” stance: e^1^. child (The garden of the fugitives, Regio I, Insula 21) and e^2^. left foot adult male (outdoor victim, XIX sec. findings); “*pugilistic attitude*” stance: a^3^. child (House of the gold bracelet) and f. adult male (outdoor victim, XIX sec. findings). Examination of the corpses' posture suggests the complete absence of any mechanical effect and an instantaneous death followed by sudden muscles contraction (cadaveric spam) due to the heat-shock induced by the PDC, as also testified by hyperflexion of hands and feet toes (flexor reflex) (e^1^, e^2^).

**Figure 3:**
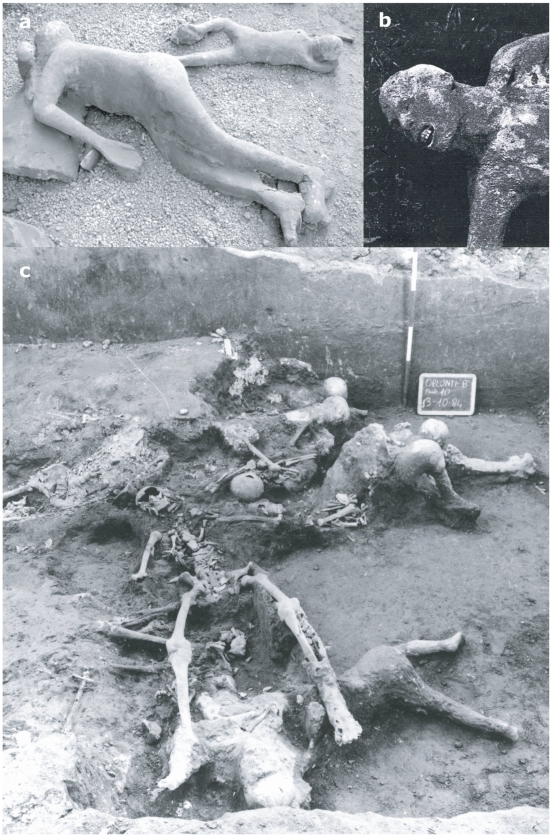
Human victims of the 79 AD eruption at Pompeii and Oplontis. a. Cast of adult woman, part of 13 human victims died outdoor (Pompeii, Garden of Fugitives) (14), possibly showing some evidence of minor mechanical impact; b. cast of adult male, part of a group of 21 victims found outdoor (Pompeii, Porta Nola), with evidence of exposure to high temperature typical of fire victims or lethality in PDCs; c. the group of human victims found in the Villa B at *Oplontis*, partially just skeletons and to some extent casts.

The primary postures include:

- “*life-like*” stance: victims that appear in suspended action (Figure 2a^1^, a^2^, b, c, 3b).
- “*sleep-like*” stance: victims laying on their back, on their right or left side in an apparent relaxed posture (Figure 2a^3^, d).
- “*impact-like*” stance: victims showing corpse displacement and/or rupture of body elements (Figure 3a).
  The secondary postures include:
- “*limb contraction*” stance: iperflexion of hands and feet (Figure 2e^1^, e^2^).
- “*pugilistic attitude*” stance: limb flexures that result from dehydration and shortening of tendons and muscles (Figure 2f).

Postures types e. and f. are generally observed as secondary effects in victims exposed to extreme heat (at least 200–300°C) [1], [16] (Figure 3b). The *pugilistic attitude* was erroneously thought to be the victim's attempt for self-defence by previous authors [15].

Similarly in Pompeii and surroundings most of the victims are typically frozen in suspended actions (73% life-like stance, 27% sleep-like stance), showing as well as limb contraction (76%) and a large number of corpses presenting the pugilistic attitude (64%). Even if different postures often coexist in the same victims group, the prevalence of people frozen in suspended actions (life-like stance) is univocally indicative of a condition known as cadaveric spasm. In contrast, postures indicative of mechanical impact effects on victims both inside and outside buildings are extremely rare (2.1%). These evidence confirm that dynamic overpressure was generally below the human lethal threshold as well as their partial or total entrapment into the current (about 2000 Pa), according with the results of our numerical modeling of PDC.

Cadaveric spasm is a rare but diagnostic form of instantaneous muscular stiffening associated with instant violent death, which crystallizes the last activity one did prior to death [17]. Such instant rigor prevents the ordinary onset of muscular relaxation immediately after death, thus avoiding any further substantial body posture modification. The presence of this stance is indicative that people was alive at the time of posture arrest and its widespread occurrence is a key evidence that all victims groups were exposed to the same lethal conditions. Cadaveric spasm commonly involves groups of muscles and only exceptionally the entire body. This last condition is described in battle situations [18], due to the exposure of victims to extreme heat. The predominance of this rare feature in Pompeii victims points to an instant death due to heat exposure.

However, since thermal human survival threshold for death has been inferred at 200°C [19], in order to verify if such condition affected the Vesuvius victims we investigated the evidence of thermally induced modifications in bones of human victims as well of a group of horses found within the Pompeii ash deposits. Therefore, we carried out macroscopic, light microscopy, histochemical and scanning electron microscopy (SEM) analyses to assess the importance of thermal effects of pyroclastic surges on casualties.

**Figure 4:**
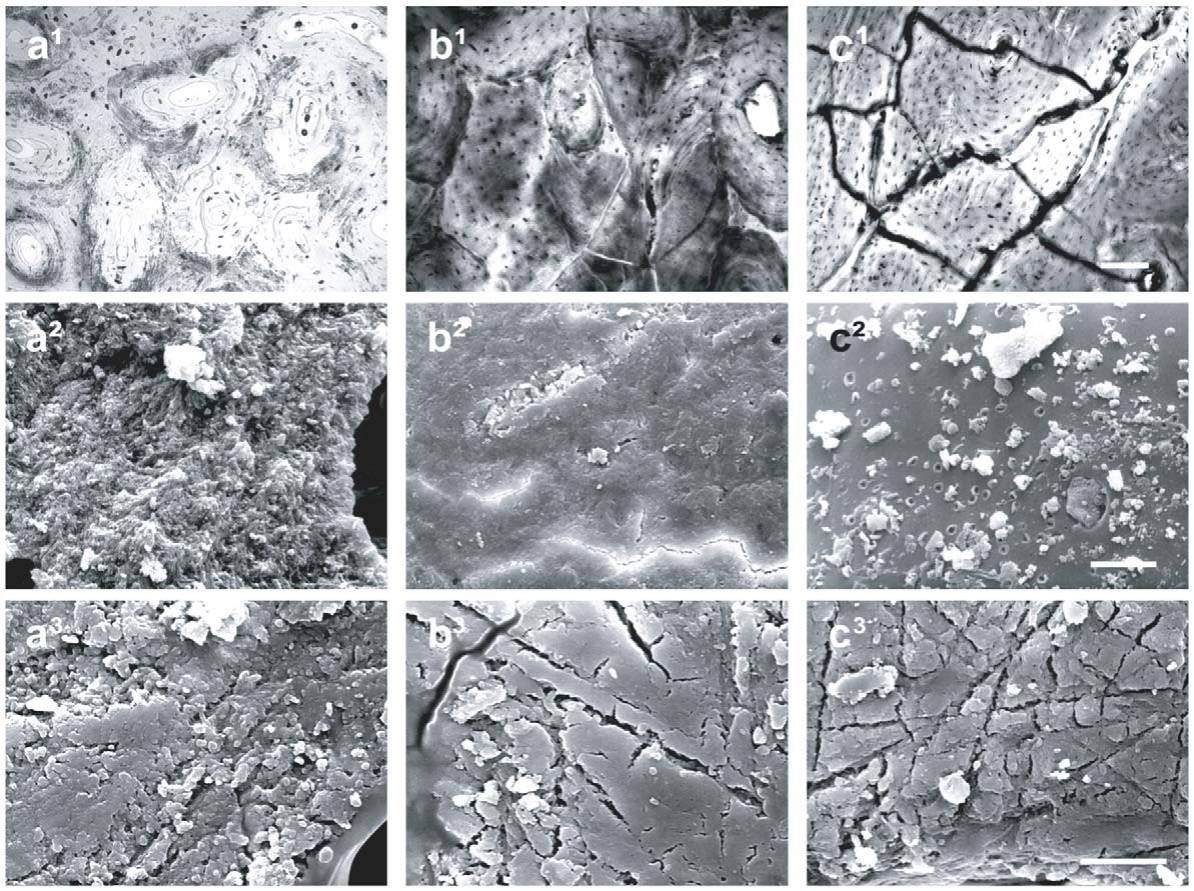
Thermal modifications in human victims bones and in recent human bones heated in laboratory. Adult bone victims analyzed with a light microscope (scale bar 100 µm) and a scanning electron microscope (scale bar 10 µm, 1700×): Femur from Pompeii showing linear cracking (a^1^) and an intact ultrastructure (a^2^); radius from *Herculaneum* showing both linear and polygonal cracking (b^1^) and incipient recrystallization (b^2^); fibula from *Oplontis* characterized by extreme polygonal cracking (c^1^) and advanced recrystallization (c^2^). SEM images of recent adult human hand phalanx heated to 200°C (a^3^), 500°C (b^3^) and 800°C (c^3^) (scale bar 5 µm, 2500×).

**Figure 5:**
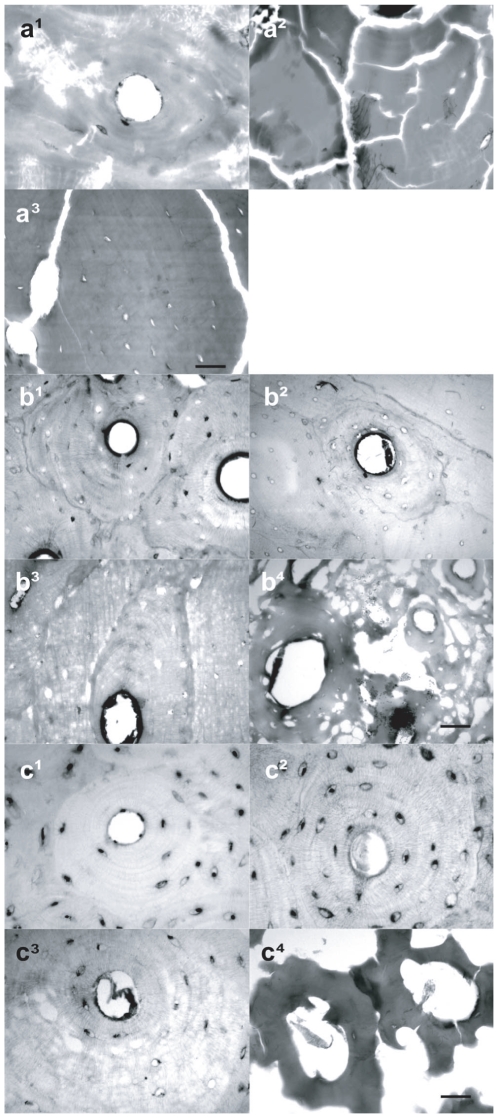
Cryostat cross-sections of decalcified ancient and heated recent human and horse bones stained with thionin. a^1^. Pompeii femur, adult; a^2^. *Herculaneum* humerus, adult; a^3^. *Oplontis* femur, adult (scale bar, 30 µm). Recent adult human phalanx exposed at 150°C (b^1^), 200°C (b^2^), 250°C (^b3^) and 300°C (b^4^) (scale bar, 50 µm); recent adult horse (*Equus caballus*) femur exposed at 150°C (c^1^), 200°C (c^2^), 250°C (c^3^) and 300°C (c^4^) (scale bar, 28 µm). In the Pompeii sample (a^1^), some osteocyte nuclei and cement lines are clearly visualized. In *Herculaneum* (a^2^) and *Oplontis* (a^3^) osteocyte nuclei are not recognizable, while microstructural damage consisting in marked linear and polygonal cracking is apparent. In both stained series of heated recent human (b^1^–b^4^) and horse (c^1^–c^4^) bone samples, DNA is clearly evident in osteocyte lacunae up to 250°C and likely persistent up to 300°C.

The bones of the Pompeii victims show colour variations ranging from natural bone colour to pale yellow as well as evidence of linear microcracking at the interosteonic level (Figure 4a^1^). All these features are indicative of exposure to high temperature [20], [21]. Additionally, our analyses by thionin staining and SEM observations reveal DNA preservation (Figure 5a^1^) and an intact bone ultrastructure (Figure 4a^2^).

**Figure 6:**
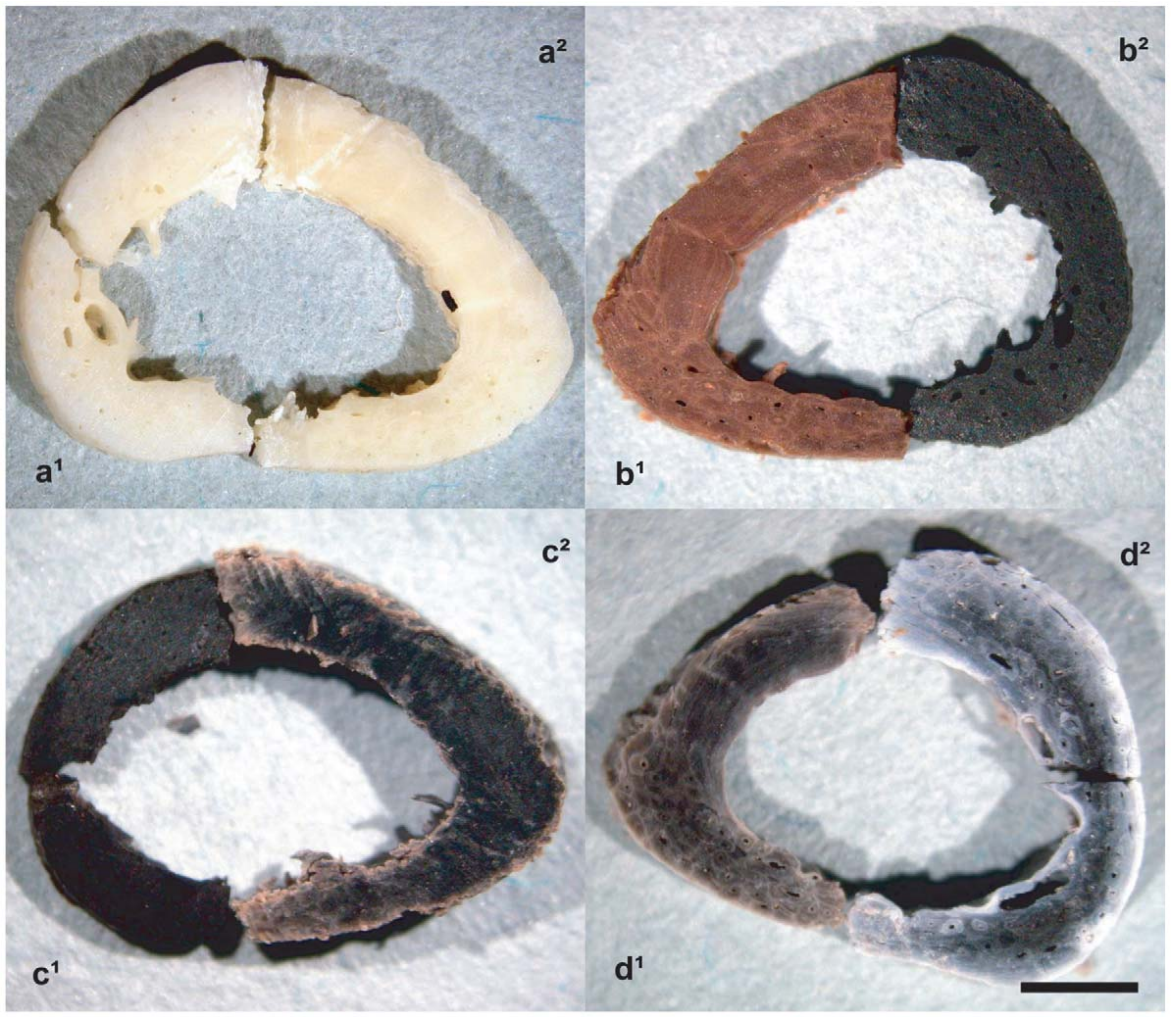
Colour features of recent human bones (adult phalanx) heated in laboratory from 100°C to 800°C. a^1^. 100°C, natural bone colour, light grey 25YR 8/1; a^2^. 200°C, pale yellow 25YR 8/3; b^1^. 300°C, bright brown 2.5YR 5/8; b^2^. 400°C, reddish black 2.5YR 1.7/1; c^1^. 500°C, black N 2/0; c^2^. 600°C, dark grey N 3/0 and pale reddish 2.5YR 7/3; d^1^. 700°C, brownish grey 7.5Y 4/1 and 5/1; d^2^. 800°C, grey N 6/0 and greyish white N 8/0 (scale bar, 2.0 mm). Bone colours are based on Munsell (1954) soil colour chart (for details on rates, limits and time of exposure see tab. S2).

**Figure 7:**
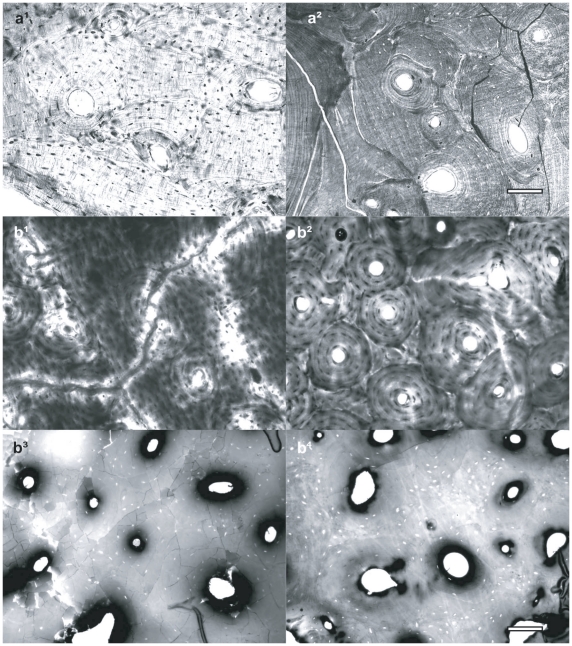
Cross ground sections of undecalcified recent human and horse bone samples heated in laboratory. Adult phalanx samples after exposure at 200°C (a^1^) and 300°C (a^2^). At 200°C the bone microstructure is well preserved, with some linear microcracking, while at 300°C it shows an evident pattern of linear as well as moderately polygonal cracking. Recent adult horse femur exposed at 200°C (b^1^), 300°C (b^2^), 400°C (b^3^) and 500°C (b^4^). At 200°C and 300°C, the well preserved bone histology shows linear microcracking. At 400°C, the bone matrix reveals an high degree of polygonal cracking and partial vanishing of the lamellar structure. At 500°C, only osteons and osteocyte lacunae are still visible, while lamellae are completely vanished. After 500°C, the increased friability of bone samples resulting in the rapid disintegration of specimen on the grinding plate did not allow to further obtain ground sections (scale bar, 100 µm).

To better constrain the range of temperature of bone modification, we heated recent human and horse (*Equus caballus*) bone samples to temperatures that range from 100° to 800°C (see data in Table 4), which are typical conditions for PDCs. At 200°C, 300°C, 400–500°C, 600–700°C and 800°C bones progressively exhibit pale yellow, bright brown, black, dark-brownish grey and light grey-white colour [10] (Figure 6), respectively, and structural microcracks increase from a linear to a polygonal pattern (Figure 7). At 500–800°C the basic bone structure recrystallizes into irregular globules (Figure 4b^3^, c^3^).

**Table 4:** Analytical data of laboratory heating experiments on recent human and fresh animal (*Equus caballus*) bones.

Sample	T°C	Rate	°C/m	Limit	Time	Colour
Human phalanx	100	1^a^	100	100°C	60″	light grey
Adult		1^b^	100	100°C	30″	25YR 8/1
Horse femur	100	1^a^	100	100°C	60″	light grey
Adult		1^b^	100	100°C	180″	25YR 8/1
Human phalanx	200	1^a^	100	100°C	60″	Pale yellow
Adult		1^b^	100	200°C	30″	25YR 8/3
Horse femur	200	1^a^	100	100°C	60″	light yellow orange
Adult		1^b^	100	200°C	180″	10YR 8/3
Human phalanx	300	1^a^	100	100°C	60″	bright brown
Adult		1^b^	100	300°C	30″	2.5YR 5/8
Horse femur	300	1^a^	100	100°C	60″	reddish brown
Adult		1^b^	100	300°C	180″	5YR 4/6
Human phalanx	400	1^a^	100	100°C	60″	reddish black
Adult		1^b^	100	400°C	30″	2.5YR 1.7/1
Horse femur	400	1^a^	100	100°C	60″	black
Adult		1^b^	100	400°C	180″	N 2/0
Human phalanx	500	1^a^	100	100°C	60″	black
Adult		1^b^	100	500°C	30″	N 2/0
Horse femur	500	1^a^	100	100°C	60″	black
Adult		1^b^	100	500°C	180″	N 2/0
Human phalanx	600	1^a^	100	100°C	60″	dark grey N 3/0
Adult		1^b^	100	600°C	30″	pale reddish 2.5YR 7/3
Horse femur	600	1^a^	100	100°C	60″	dark grey
Adult		1^b^	100	600°C	180″	N 3/0
Human phalanx	700	1^a^	100	100°C	60″	brownish grey
Adult		1^b^	100	700°C	30″	7.5Y 4/1 - 5/1
Horse femur	700	1^a^	100	100°C	60″	dark grey
Adult		1^b^	100	700°C	180″	N 3/0
Human phalanx	800	1^a^	100	100°C	60″	grey N 6/0
Adult		1^b^	100	800°C	30″	greyish white N 8/0
Horse femur	800	1^a^	100	100°C	60″	dark grey N 3/0
adult		1^b^	100	800°C	180″	greyish white N 8/0

Our comparative histochemical analyses on the DNA within osteocyte lacunae of heated human (Figure 5b^1^–b^4^) and horse (Figure 5c^1^–c^4^) bones reveal that DNA is persistent up to 300°C, whereas it is undetectable at higher temperatures. A comparison of macroscopic, microscopic and ultrastructural bone features of Pompeii victims (Figure 4a^1^, a^2^) with results of our laboratory experiments (Figure 4a^3^–c^3^, Figure 7) suggest that the ancient bones were exposed to temperatures of 250–300°C [20]. Thus indicating that these values likely correspond to minumum pyroclastic surge temperature due to the buffering effects of soft tissues.

A parallel analysis was conducted on victims from the town of *Herculaneum* and the suburban *Oplontis*, both of which lie at about 7 kilometres from Vesuvius. The remains of victims here consist exclusively of skeletons (*Herculaneum*) or skeletons with only partial body imprint in the ash (*Oplontis*, Figure 3c). The analyzed specimens from *Herculaneum* and *Oplontis* show bone colours ranging from black to grey-white, linear to polygonal microcracks (Figure 4a^1^–c^1^) and incipient to high recrystallization (Figure 4b^2^, c^2^), as well as complete DNA degradation (Figure 5a^2^, a^3^). In contrast to Pompeii but similar to *Herculaneum* [16] several victims at *Oplontis* show skull explosion, as testified by clear-cut fractures resulting from intracranial overpressure induced by exposure of the corpses to very high temperature. These features suggest temperatures of ca 500°C in *Herculaneum*, which matches well with previous supporting evidence [16], and a temperature of ca 600°C in *Oplontis* [22].

The total thermal energy, which is directly related to the deposit thickness and the emplacement temperature, is then lower at Pompeii than in *Oplontis* and *Herculaneum*. Therefore heat was enough for sudden and complete vaporization of soft tissues of the victims at *Herculaneum* and *Oplontis*, where the flesh was suddenly replaced by the ash, but was insufficient at Pompeii. This account for the nearly perfect preservation of the entire body imprint (plaster casts) in the ash as a consequence of the delayed disappearance of flesh of these bodies.

## Discussion

Our overall results indicate that S4 pyroclastic surge crossed Pompeii as a dilute poorly-energetic dusty gas cloud and emplaced suddenly in response to horizontal velocity decay and turbulence dumping, approaching its termination just next the southern town walls. Due to low density and velocity, its dynamic overpressure was low with negligible effects on the structures and people. The PDC had a concentration of inhalable ash particles (<100 microns) in the order of 0.1÷0.7 kg/m^3^, the lower values commonly observed at the head of dilute PDCs, and temperature range between 250 and 600°C. These values are consistent with the pyroclastic surge temperatures computed for the marginal zone of PDCs derived from general numerical modeling of Plinian column collapse [23].

An independent verification that PDC temperatures in Pompeii exceeded 250°C is the melting of silverware solder [24]. In Roman times this material was made with a lead/tin alloy like *Tertiarium* (Pb-Sn 2∶1) [25], which has a melting point of ca 250°C. A temperature of 250°–300°C was also high enough to char wood objects, vegetal material and food [26] but was unable to affect glass [24], that is preserved intact in the ash deposits.

Nevertheless, the exposure time of the victims to high temperature and dusty gas was very short as resulting from lasting passage of S4 surge in the range of 30÷1.5×10^2^ seconds. This result is crucial being the capability of PDCs to cause death and injury not only depending on their physical conditions but also on the exposure time. The passage time is consistent with the inferred lethal time for temperatures in the range of 250°–600°C evaluated from ca 10 to ca 10^2^ seconds [27]. Notably, such a time lapse is insufficient to cause asphyxia that would require an exposure time of several minutes, thus indicating that people would be able to survive to suffocation in 0.5 to 2.5 minutes of the S4 surge cloud passage [1]. Nevertheless, the calculated concentration of inhalable ash in the PDC approached the survival condition in the order of ca. 0.1 kg/m^3^. Consistently, the widespread occurrence of primary life-like postures (cadaveric spasm) in the victims is only compatible with an instantaneous death, while exclude the longer agony and the final floppy posture that characterize suffocation.

Finally, contrary to previous hypotheses, our findings based on the interdisciplinary volcanological and bio-anthropological study of the deposits and victims of the 79 AD Plinian eruption reveal that even at the extreme periphery of the S4 surge neither asphyxia nor impact force but heat caused the deaths. Actually, while impact force and exposure time to dusty gas dropped below lethal conditions, the pyroclastic cloud retained its high temperature thus being the main cause of instantaneous mortality for the Vesuvius area inhabitants, including people who were sheltered within buildings as far as in Pompeii. Definitely, a group of indoor victims found at Muregine, within the S4 PDC limit about half kilometer south-east of Pompeii walls, suggests that even an extremely short exposure to the pyroclastic surge in the order of seconds to a few tents of seconds was lethal.

These facts and the evidence that the late, most powerful 79 AD PDCs reached distance exceeding 20 kilometres from the vent and the findings of several scattered groups of victims in Roman villas even as far as at least 15 kilometres in *Stabiae* highlight the need to strengthen the emergency plans for Vesuvius and other similar explosive volcanoes considering long-distance thermal effects even at the extreme PDCs periphery as primary cause of fatalities.
